# Relationship Between Forced Vital Capacity and Framingham Cardiovascular Risk Score Beyond the Presence of Metabolic Syndrome: The Fourth Korea National Health and Nutrition Examination Survey

**DOI:** 10.1097/MD.0000000000002089

**Published:** 2015-10-30

**Authors:** Hyung Koo Kang, Hye Yun Park, Byeong-Ho Jeong, Won-Jung Koh, Seong Yong Lim

**Affiliations:** From the Division of Pulmonary and Critical Care Medicine, Department of Medicine, Samsung Medical Center, Sungkyunkwan University School of Medicine, Seoul, South Korea (HKK, HYP, BHJ, WJK); and Division of Pulmonary and Critical Care Medicine, Department of Medicine, Kangbuk Samsung Hospital, Sungkyunkwan University School of Medicine, Seoul, South Korea (SYL).

## Abstract

Impaired lung function is a risk factor for cardiovascular (CV) events. However, it has not been well established whether FVC reduction even within normal range is associated with cardiovascular disease (CVD) risk and whether reduced FVC is an independent relationship of CVD irrespective of metabolic syndrome. Thus, we aimed to explore the relationship between FVC and CV-event risk using the FRS beyond the presence of metabolic syndrome or abdominal obesity in a representative Korean population based on data from the nationwide Korea National Health and Nutrition Examination Survey (KNHANES IV).

The study population included 9688 subjects ≥ 30 years of age with no previous diagnosis of CVD and obstructive lung disease. Using a logistic regression model and area under the curve (AUC) analysis, we evaluated the relationship between FVC quintiles and CV-event risk using the Framingham Risk Score (FRS; ≥ 10% or ≥ 20%). In addition, we examined the effect of FVC on CV-event risk based on the presence of metabolic syndrome (MetS) and abdominal obesity.

After adjusting for covariates, comparison of subjects in the lowest FVC (% pred) quintile (Q1) with those in the highest quintile (Q5) yielded an odds ratio (OR) of 2.27 (95% CI, 1.91–2.71) for intermediate and high risk, and 2.89 (95% CI, 2.31–3.61) for high risk. The ORs for cardiovascular risk using FRS also increased irrespective of the presence of abdominal obesity and MetS without significant interaction. Furthermore, the addition of FVC status to MetS status and abdominal obesity status significantly increased the AUC of the model predicting CV-event risk (*P* < 0.001 and *P* < 0.001).

Our study demonstrates that FVC is inversely associated with 10-year CV-event risk, irrespective of MetS and abdominal obesity in the general population without obstructive lung disease. Furthermore, the addition of FVC to MetS or abdominal obesity increased prediction of CVD event risks, implying a potential role of FVC to predict CV events.

## INTRODUCTION

Cardiovascular diseases (CVDs), including coronary, cerebrovascular, peripheral arterial disease, and heart failure, are leading causes of morbidity and mortality worldwide.^1,2^ It is well recognized that risk factors such as hypertension, diabetes mellitus (DM), high cholesterol levels, and obesity contribute to the development of CVD.^3–7^ Metabolic syndrome (MetS) is the clustering of these cardiovascular risk factors including abdominal obesity, dyslipidemia, hypertension, insulin resistance, and prothrombotic states that can promote CVD.^8,9^

Although several risk scores have been used for predicting CVD risk,^10–12^ the Framingham Risk Score (FRS) is the most popular global risk algorithm for estimating 10-year cardiovascular (CV)-event risk. The FRS uses multiple risk factors such as age, sex, smoking history, systolic blood pressure, total cholesterol levels, high-density lipoprotein (HDL), cholesterol levels, and diabetes status in individuals not previously diagnosed with CVD.^13,14^

Impaired lung function, such as reduced forced expiratory volume in 1 s (FEV_1_) and forced vital capacity (FVC), is another risk factor for CVD morbidity and mortality.^15–18^ Several previous studies have shown that both obstructive and restrictive lung function impairment have a positive independent relationship with MetS, with abdominal obesity playing a critical role.^19–21^ However, it has not been well established whether FVC reduction even within normal range is associated with cardiovascular disease (CVD) risk. In addition, although chronic obstructive pulmonary disease (COPD) representing reduced FEV_1_ has been shown to be a risk factor for CVD^16^ and MetS,^22^ there have been few population-based studies which reported the relationship between FVC and FRS-assessed future CVD risk after adequately adjusting the covariates such as the presence of MetS or abdominal obesity, both of which have been found to play a role in the development of CVD.

Therefore, in the present study, we aimed to determine whether there exists an independent relationship between FVC and CV-event risk using the FRS beyond the presence of metabolic syndrome or abdominal obesity in a representative Korean population based on data from the nationwide Korea National Health and Nutrition Examination Survey (KNHANES).

## METHODS

### Subjects

KNHANES is a cross-sectional, nationwide, population-based health survey that uses complex, stratified, multistage cluster sampling.^23^ We used KNHANES to select a representative nationwide sample of the noninstitutionalized Korean population conducted by the Korea Centers for Disease Control and Prevention (KCDC). The KHANES has been conducted periodically since 1998, and a fourth survey was conducted between July 2007 and December 2009. We performed a retrospective review of the data from the fourth KNHANES.

Among 24,871 potential subjects, a total of 9688 subjects were included in our study. Subjects <30 years of age (n = 8969) were excluded, as an FRS could not be calculated for this population.^13^ We also excluded subjects (n = 6213) who had been previously diagnosed with myocardial infarction, angina pectoris, stroke, asthma or COPD, and/or had an obstructive pattern in spirometry (FEV_1_/FVC < 70%).^24^ Lastly, 1 subject with missing values of height and weight was excluded.

The KNHANES received ethical approval by the Institutional Review Board of the KCDC (IRB No: 2007-02-CON-04-P, 2008-04EXP-01-C, 2009-01CON-03-2C), and written consent was obtained from all of the participants. In addition, the study was conducted in accordance with the ethical principles of the Declaration of Helsinki.

### Measurements

#### Anthropometric Measurements and Blood Tests

Height, weight, waist circumference (WC), systolic and diastolic blood pressures (BP) were measured. Blood pressure (BP) was measured 3 times by nurses trained in mercury sphygmomanometer use, with the participants in a seated position after a 5 min rest. The final BP value was obtained by averaging the values of the second and third BP measurements. Height and weight were measured by using an automatic scale, and the body mass index (BMI, kg/m^2^) was calculated by dividing weight (kg) by the squared value of height (m). Because obesity is defined as BMI ≥ 25 kg/m^2^ in Asian populations,^25^ BMI in our study was defined by a cut-off point of 25 kg/ m^2^ indicative of obesity. Waist circumference was measured at the portion of the trunk located midway between the lower costal margin and the iliac crest while the patient was standing.

Blood samples were obtained after 12 h of fasting. Total cholesterol, triglyceride levels, and HDL cholesterol levels were enzymatically measured, whereas low-density lipoprotein (LDL) cholesterol levels were calculated using the Friedewald equation^26^:

(LDL-cholesterol) = (total cholesterol) – (HDL-cholesterol) – (triglyceride)/5.

White blood cell (WBC) counts and ferritin levels were measured automatically with laserflow cytometry and immunoradiometric assay, respectively.

Medical and social histories were reviewed. Comorbidities, duration of education, physical activity, and smoking status were obtained by questionnaire. Asthma and COPD were defined by a patient-reported physician's diagnosis Hypertension and DM were defined by a patient-reported physician's diagnosis, or the use of antihypertensive and hypoglycemic agents, respectively. Duration of education was divided into 4 groups as the subjects’ highest level of education. In addition, subjects were questioned the level of physical activity in the following 3 categories: (a) Did you walk for at least 30 min, 5 times on a recent week? (b) Did you perform moderate physical activity for at least 30 min, 5 times on a recent week? and (c) Did you perform vigorous physical activity for at least 20 min, 3 times on a recent week? The physical activity was divided in 3 different levels: (a) low, subjects who did not belong in any of 3 categories; (b) moderate, subjects who belong to 1 of the 3 categories; and (c) high, subjects who belong to 2 or 3 categories.^27^ Smoking status was categorized as lifetime nonsmoker and smoker.

#### Framingham Risk Score Calculation and Cardiovascular Risk Stratification

FRS was calculated based on risk factors including gender, age, total cholesterol, HDL cholesterol, systolic BP, treatment for hypertension, smoking status, and DM status.^13^ The Framingham risk groups (taking all risk factors into account) were defined by risk percentages (low < 10%, intermediate 10–20%, high > 20%).

#### Lung Function Measurement

Spirometry was performed as recommended by the American Thoracic Society.^28^ We analyzed only data from subjects with 2 or more acceptable spirometry performances. Absolute values of FVC and FEV_1_ were obtained, and the percentage predicted values (% pred) for FEV_1_ and FVC were calculated from the following equations obtained in a representative Korean sample:^29^

Predicted FVC = −4.8434 − (0.00008633 × age2 [years]) + (0.05292 × height [cm]) + (0.01095 × weight [kg])

Predicted FEV_1_ = −3.4132 − (0.0002484 × age2 [years]) + (0.04578 × height [cm])

We analyzed the patients according to the quintile of FVC (% pred) excluding patients exhibiting an obstructive pattern (FEV_1_/FVC < 70%). To evaluate the relationship between FVC (% pred) and Framingham risk, we divided subjects into quintiles based on FVC (% pred) (quintile 1[Q1], <84%; quintile 2[Q2], 84–90%; quintile 3[Q3], 91–95%; quintile 4[Q4], 96–102%; quintile 5[Q5], ≥103%).

#### Metabolic Syndrome (MetS) Diagnosis

Metabolic syndrome was defined by the American Heart Association/National Heart, Lung, and Blood institute (AHA/NHLBI), whereas WC was defined by the Western Pacific Region of WHO for obesity (WPRO) criteria.^30^ In our population, the diagnosis of MetS was made if the patient had 3 or more of the following risk factors: (a) abdominal obesity (WC ≥ 90 cm), (b) BP ≥ 130/85 mm Hg, (c) HDL cholesterol level < 40 mg/dL (1.0 mmol/L), (d) triglyceride level ≥ 150 mg/dL (1.7 mmol/L), (e) fasting glucose level ≥ 100 mg/dL (5.6 mmol/L).

### Statistical Analysis

Baseline characteristics across quintiles were compared using ANOVA for continuous variables and a Cochran-Armitage trend test for dichotomous variables. A logistic regression model was used to assess the relationship between FVC (% pred) quintiles and CV-event risk using FRS (≥ 10% or ≥ 20%) using Q5 (ie, the group with the highest FVC [% pred]) as the referent. In multivariate analysis, 3 models were constructed. Model 1 was adjusted for sex, smoking, education, abdominal obesity, obesity, medical history of DM, and hypertension. Model 2 was additionally adjusted for physical activity, white blood cell counts, LDL-cholesterol, and serum ferritin. Model 3 included the presence of metabolic syndrome as a confounding factor, but excluded abdominal obesity, obesity, and medical history of DM, and hypertension. Furthermore, to maximize evaluation of the effect of FVC on CV-event risk, FVC values were dichotomized into an FVC-Q1 group and an FVC-Q2-5 group. In the final model, we analyzed dichotomized FVC-MetS interaction and dichotomized FVC-abdominal obesity for CV-event risk. Area under the curve (AUC) or the C statistic was performed to evaluate CV-event risk beyond MetS and abdominal obesity. All statistical analyses were performed using IMB SPSS Statistics for windows, version 22.0 (Armonk, NY) and STATA 13 (STATA, College station, TX).

## RESULTS

### Clinical Characteristics by FVC Quintile

**FIGURE 1:**
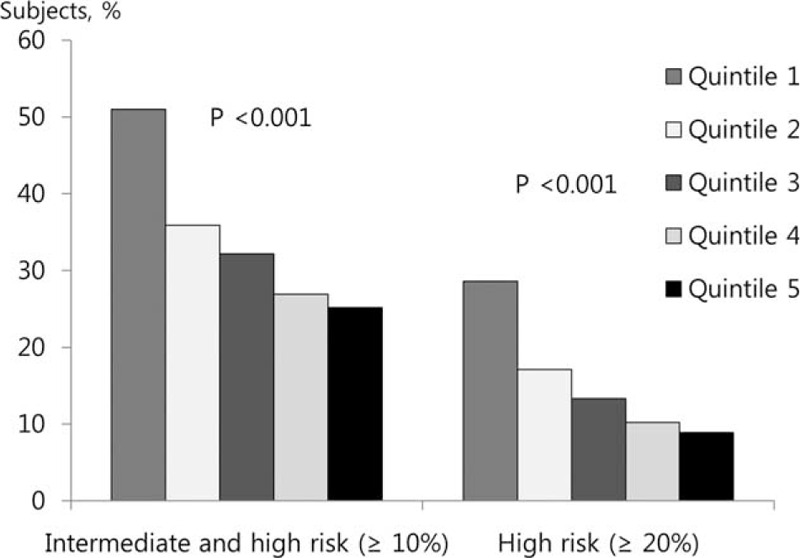
Proportions of subjects with intermediate or high 10-yr CV-event risk by FVC (% pred) quintile.CV = cardiovascular, FVC = forced vital capacity.

Clinical characteristics of the study population based on FVC (% pred) quintile are presented in Table 1. Waist circumference, prevalence of WC-defined abdominal obesity, BMI, prevalence of BMI-defined obesity, DM, hypertension, and MetS were inversely associated with FVC (% pred), whereas the physical activity level was similar across FVC (% pred) quintiles. The mean values of white blood cells counts, total cholesterol, triglycerides, and ferritin had a significant inverse relationship with FVC (% pred), whereas HDL cholesterol had a significant positive relationship with FVC (% pred). Proportions of subjects with intermediate or high 10-year CV-event risk stratified by the FRS group increased in a stepwise manner as FVC decreased from highest (Q5) to lowest (Q1) (Fig. 1).

**TABLE 1:**
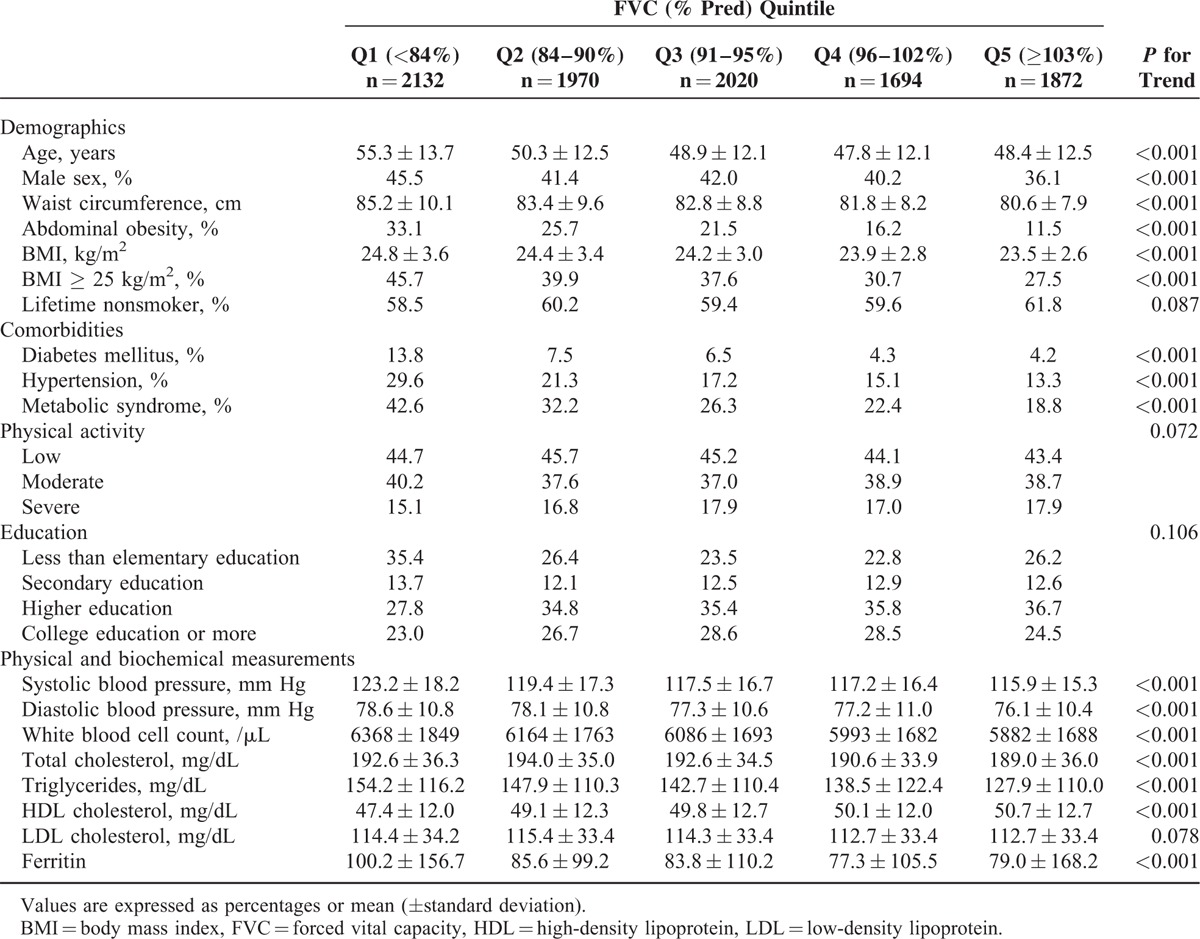
Comparisons of Demographics, Comorbidities, and Physical and Biochemical Measurements According to the Quintiles of FVC % Predicted Value

### Framingham Risk by FVC Quintile

The relationships between FVC (% pred) and FRS is shown in Table 2. When the highest quintile (Q5) of FVC (% pred) was considered the referent, the odds ratio (OR) for the FRS ≥ 10% and ≥ 20% significantly increased as FVC (% pred) quintile decreased (*P* for trend < 0.001). The ORs in the lowest FVC (% pred) quintile (Q1) for FRS ≥ 10% and ≥ 20% were 3.09 (95% CI, 2.69–3.54) and 4.08 (95% CI, 3.39–4.92), respectively. This association persisted even after adjustment for covariates (Table 2).

**TABLE 2:**
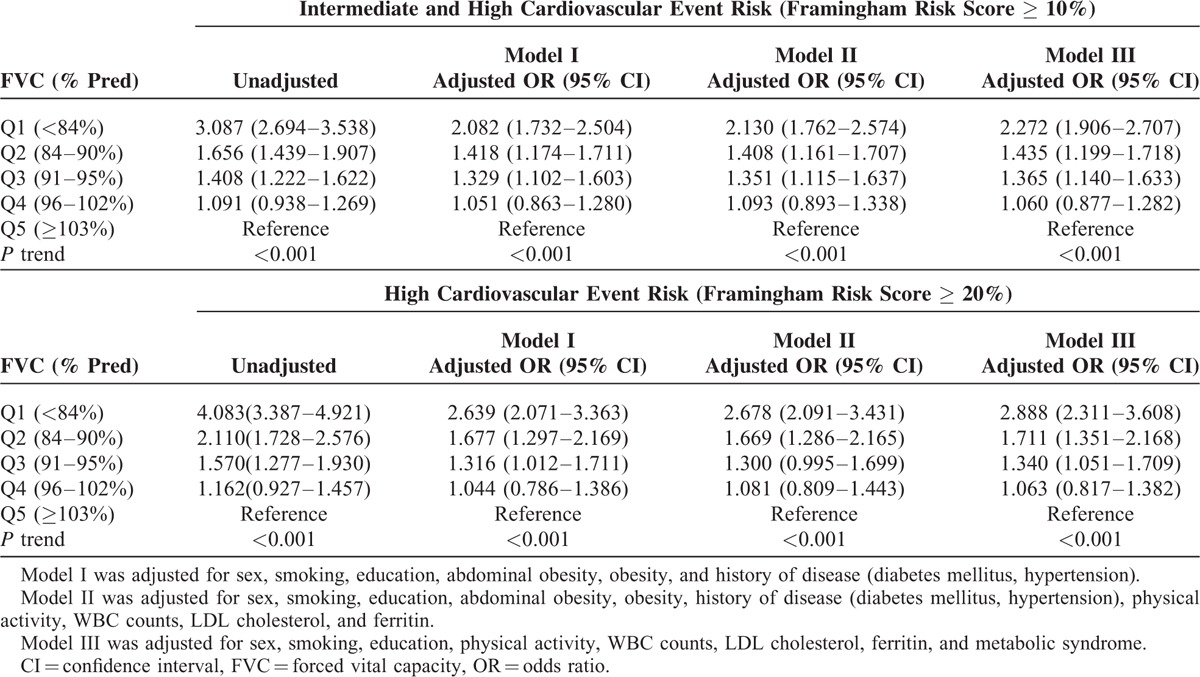
Ten-Year Cardiovascular Event Risk According to FVC (% pred) in Patients without Obstructive Lung Disease

### Framingham Risk by FVC % Quintile Based on the Presence of Metabolic Syndrome and Abdominal Obesity

**FIGURE 2:**
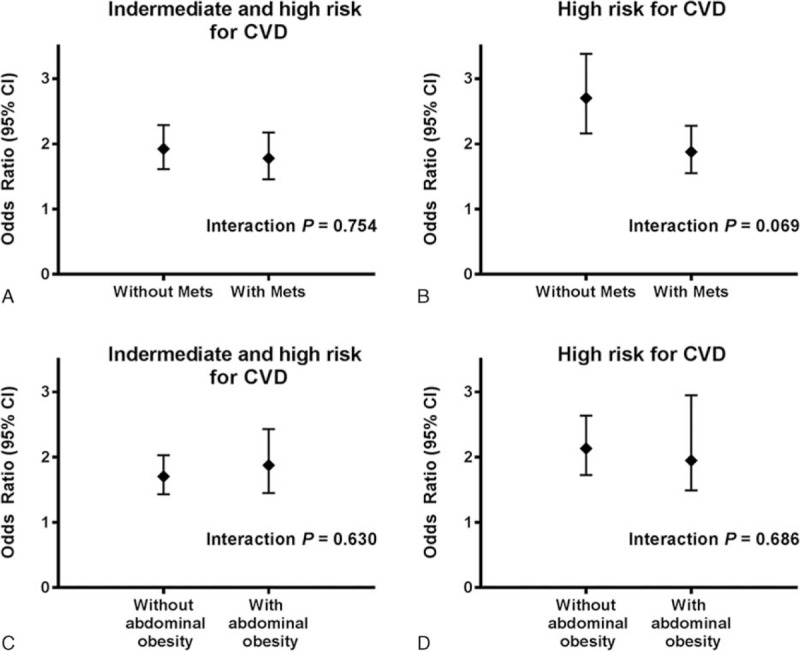
Odds ratio for FRS according to FVC (% pred) based on the presence of metabolic syndrome (A,B) and abdominal obesity (C,D). FRS = Framingham Risk Score.

The ORs for CV-event risk ≥ 10% in subjects with the lowest FVC values (Q1) with and without MetS were 1.78 (95% CI, 1.46–2.18) and 1.92 (95% CI, 1.62–2.29), respectively, compared to the other groups (Q2, Q3, Q4, and Q5) after adjustment for covariates including sex, smoking, education level, physical activity, white blood cell counts, LDL cholesterol levels, and serum ferritin. The ORs for CV-event risk ≥ 20% in subjects with the lowest FVC values (Q1) with and without MetS were 1.88 (95% CI, 1.55–2.27) and 2.70 (95% CI, 2.16–3.39), respectively, compared to the other groups (Q2, Q3, Q4, and Q5). However, the *P* values for the interaction between the presence of MetS and FVC quintile for FRS ≥ 10% and ≥ 20% were 0.754 and 0.069, respectively (Fig. 2).

The ORs for CV-event risk ≥ 10% in subjects with the lowest FVC values (Q1) with and without abdominal obesity were 1.88 (95% CI, 1.45–2.43) and 1.71 (95% CI, 1.43–2.03) in the adjusted model, compared to the other groups (Q2, Q3, Q4, and Q5). The ORs for CV-event risk ≥ 20% in subjects with the lowest FVC values (Q1) with and without abdominal obesity were 1.95 (95% CI, 1.49–2.95) and 2.13 (95% CI, 1.73–2.64), respectively, compared to the other groups (Q2, Q3, Q4, and Q5). However, the interaction between the presence of abdominal obesity and FVC did not achieve statistical significance after adjustment for covariates including sex, smoking, education level, obesity, comorbidities, physical activity level, WBC counts, LDL cholesterol, and serum ferritin (Fig. 2).

Metabolic syndrome status for FRS ≥ 10% and ≥ 20% yielded an AUC of 0.685 (95% CI, 0.675–0.694) and 0.684 (95% CI, 0.671–0.698), respectively. The addition of FVC status significantly increased the AUC of the model to 0.714 (*P* < 0.0001) and 0.723 (*P* < 0.0001), respectively. Abdominal obesity status for FRS ≥ 10% and ≥ 20% yielded an AUC of 0.684 (95% CI, 0.673–0.695) and 0.678 (95% CI, 0.664–0.692), respectively. The addition of FVC status significantly increased the AUC of this model to 0.699 (*P* < 0.0001) and 0.703 (*P* < 0.0001), respectively, providing additional discrimination for CV-event risk beyond MetS and abdominal obesity (Table 3).

**TABLE 3:**
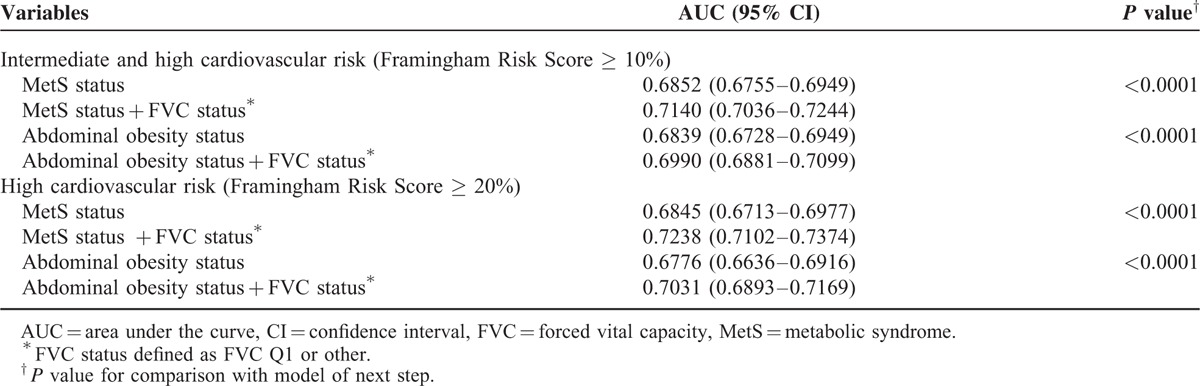
Area under the Curve Values from the Presence of Metabolic Syndrome and Abdominal Obesity Predict Relationship between Framingham Risk Score and FVC

## DISCUSSION

According to the results of our study, FVC (% pred) was inversely associated with FRS-determined 10-year CVD-event risk, irrespective of central obesity and MetS, in Koreans without obstructive lung disease. Proportions of subjects in intermediate or high FRS groups gradually increased as FVC decreased from highest (Q5) to lowest (Q1). After adjusting for several factors such as sex, smoking, education level, abdominal obesity, obesity, history of disease (diabetes mellitus, hypertension), physical activity, WBC counts, LDL cholesterol, and ferritin, subjects with the lowest FVC values (Q1) had an intermediate 10-year CV-event risk approximately 2 times greater than that of subjects in the FVC reference group (Q5). Additionally, subjects with the lowest FVC values (Q1) had a high CV-event risk, nearly 3 times greater than that of subjects in the FVC reference group (Q5). The ORs for CV risk using FRS also increased irrespective of the presence of abdominal obesity and MetS. Furthermore, the addition of FVC status significantly increased the AUC of the model with MetS status or abdominal obesity status, providing additional discrimination for cardiovascular event risk beyond MetS and abdominal obesity. We found that the effect of FVC on CV-event risk is important regardless of the presence of MetS or abdominal obesity.

In our study, cardiovascular risk increased from FVC-Q3, ranging from 91% to 95% (often considered normal). This result suggests that both restrictive lung disease and reduced FVC, even within the normal range, may increase the odds of undergoing a CV-event. This observation is consistent with a previous US NHANES follow-up report by Sin et al^15^. The group identified that even a modest decline in FEV_1_ (from a mean of 109% to 88%, a value still, considered normal) was associated with a 5-fold increase in ischemic heart disease mortality. Future prospective, longitudinal studies are needed to determine whether there exists a critical FVC threshold that may predict the risk of developing CVD.

Several long-term population studies have suggested an association between FVC and CVD.^17,31–34^ Some studies have also demonstrated that decreased FVC is associated with electrocardiographic ST-T abnormalities and arterial hypertension.^35,36^ The decreased lung function has been shown to be related to arterial calcification and stiffness,^37,38^ which is linked with increased incidence of CVD. However, few studies have evaluated the relationship between FVC and CVD in Asian populations without obstructive lung disease. To date there have been no studies evaluating the effect of FVC on CV-event risk based on the presence of MetS and abdominal obesity. To the best of our knowledge, this study is the first study in Asia which evaluated the relationship between FVC and CVD risk targeted healthy individuals without obstructive lung disease using a large, representative sample of the Korean population. Even after adjusting for several factors (especially abdominal obesity and MetS, both well-known risk factors for CVD), a decrease in FVC affected the risk of CVD in both intermediate- and high-risk groups. Furthermore, the addition of FVC status provided additional discrimination for CV-event risk beyond MetS and abdominal obesity.

As a protective factor, regular physical activity is associated with reduced rates of CVD, obesity, and metabolic syndrome.^39^ Consistently, we also observed that physical activity had an inverse relationship with CV-event risk using the FRS (*P* = 0.005). However, even after adjusting for physical activity, a decrease in FVC independently affected the risk of CVD in both intermediate- and high- risk groups.

Although our study demonstrated that FRS-based CVD risk increased in a stepwise manner as FVC decreased from highest (Q5) to lowest (Q1), the mechanism underlying this association remains unclear. Several explanations behind this association have been proposed. Previous studies have suggested that abdominal obesity might be to blame. First, the mechanical effect of abdominal obesity, which is measured by WC, could influence respiratory mechanics.^21^ Abdominal obesity likely affects lung volumes without direct pulmonary obstruction,^40^ and FVC may be decreased by compression of the diaphragm, ultimately preventing lung from expansion. Second, systemic inflammation from adipose tissue may play a role in the association between FVC and FRS.^19,20^ Recent data has shown that systemic inflammation may be a causative factor,^41,42^ and systemic inflammatory markers such as C-reactive protein (CRP) and fibrinogen have been implicated in the association.^43,44^ Engstrom et al found that low FVC values were associated with high inflammation-sensitive plasma proteins (ISPs) and inflammatory markers contributed to increased incidence of CV events.^45^ In our study, we found that systemic inflammatory markers (WBC counts and ferritin) significantly increased as FVC decreased, which is consistent with previous studies. Therefore, our findings clearly support that a decrease in FVC could be related to systematic inflammation as opposed to lung compliance.

There are several limitations in our study. First, this study evaluated the risk of CVD indirectly using FRS, rather than mortality or morbidity of CVD. Future longitudinal studies are needed to verify the relationship between FRS and CVD. Second, because serum CRP was not included in the KNHNES, we were unable to adjust serum CRP, widely considered a key inflammatory marker related to decreased lung function.^46^ Instead, we adjusted WBC and ferritin, also known to be important indicators of systemic inflammation related to CVD.^47,48^

Finally, population's diverse profession could not be categorized based on physical activity, although Leischik et al showed that sedentary occupations are associated with lower HDL cholesterol, higher LDL cholesterols, and higher waist circumferences, compared with occupation with highly physical activity.^49^ The investigation of the independent relationship between FVC and CV-event risk after the adjustment of professions based on sedentary lifestyle will be necessary.

In conclusion, this study clearly demonstrated a significant association between decreased FVC and increased CV-event risk in a Korean population without obstructive lung disease, irrespective of MetS and abdominal obesity, and suggested the promising role of FVC for prediction of CV events beyond MetS or abdominal obesity.
